# Concept and Development of an Accelerated Repeated Rolling Wheel Load Simulator (ARROWS) for Fatigue Performance Characterization of Asphalt Mixture

**DOI:** 10.3390/ma14247838

**Published:** 2021-12-17

**Authors:** Zeyu Zhang, Julian Kohlmeier, Christian Schulze, Markus Oeser

**Affiliations:** Institute of Highway Engineering, RWTH Aachen University, D52074 Aachen, Germany; zeyu.zhang@isac.rwth-aachen.de (Z.Z.); schulze@isac.rwth-aachen.de (C.S.); oeser@isac.rwth-aachen.de (M.O.)

**Keywords:** asphalt mixture, fatigue performance, rolling load, laboratory test, device

## Abstract

Fatigue performance is one of the most important properties that affect the service life of asphalt mixture. Many fatigue test methods have been developed to evaluate the fatigue performance in the lab. Although these methods have contributed a lot to the fatigue performance evaluation and the development of fatigue related theory and model, their limitations should not be ignored. This paper starts by characterizing the stress state in asphalt pavement under a rolling wheel load. After that, a literature survey focusing on the experimental methods for fatigue performance evaluation is conducted. The working mechanism, applications, benefits, and limitations of each method are summarized. The literature survey results reveal that most of the lab test methods primarily focus on the fatigue performance of asphalt mixture on a material level without considering the effects of pavement structure. In addition, the stress state in the lab samples and the loading speed differ from those of asphalt mixture under rolling wheel tire load. To address these limitations, this paper proposes the concept of an innovative lab fatigue test device named Accelerated Repeated Rolling Wheel Load Simulator (ARROWS). The motivation, concept, and working mechanism of the ARROWS are introduced later in this paper. The ARROWS, which is under construction, is expected to be a feasible and effective method to simulate the repeated roll wheel load in the laboratory.

## 1. Introduction

Asphalt pavement withstands millions of rolling wheel loads after opening to traffic. The rolling of wheels on the top of the pavement brings tensile, compressive, and shear stresses to the asphalt mixture. Repeating this phenomenon over the long term creates fatigue damage, degrading the integrity of asphalt mixture by creating and growing micro-cracks or cavities. As a consequence of fatigue damage accumulation, longitudinal cracks appear on pavement surface and propagate into alligator cracks, turning asphalt pavement into essentially a gravel layer as loading continues [1]. Therefore, accurately capturing and predicting the fatigue behavior of asphalt mixture is vital to prevent asphalt pavement from premature fatigue failure. For this purpose, researchers have proposed many test technologies during the past few decades, such as 4-point bending (4PB) fatigue test method [2], and indirect tensile fatigue test (ITFT) method [3]. These laboratory test methods enable the researchers to determine the influences of inherent material properties (e.g., air voids content, involved binder type, and aggregate gradation) and external service conditions (e.g., temperature, loading frequency, and load amplitude) on the fatigue response of asphalt mixtures, and to develop theories and models concerning the fatigue behavior of asphalt materials [4,5,6]. Although it has been extensively used, the fatigue test method is still in a dynamic development process. The overall goal of these efforts is to eliminate the differences between the lab-measured and in situ observed fatigue and cracking behavior of asphalt mixture. Specifically, researchers try to optimize the experimental setup of the fatigue tests to better represent the stress state in asphalt pavement. Bending fatigue test is designed to simulate the flexural stress at the bottom of the asphalt layer, and ITFT is expected to evaluate the fatigue response of asphalt mixture under the two-dimensional stress. The load waveform, loading frequency, and test temperature are also carefully selected to simulate the realistic service condition of asphalt pavement.

Despite the fact that much work has been done, the laboratory fatigue test technologies still have great limitations in simulating the field fatigue performance of asphalt mixture. For instance, the flexural stress at the bottom of the asphalt layer of pavement is two-dimensional. Fatigue damage occurs due to the repetition of flexural stress at both longitudinal and transverse directions caused by repeated rolling load. None of the currently available fatigue test methods can simulate this phenomenon. Hence, a paper is demanded to holistically present the development, current state of art, and limitations of the fatigue test technologies. To this purpose, this paper presents a literature survey concerning the currently available fatigue test technologies in various aspects, including sample geometry, load control mode, and load frequency. Prior to the literature survey, the stress state in asphalt mixture under the rolling tire load is discussed. Based on the literature survey results, the concept of an innovative fatigue test device named ARROWS, which is the acronym of Accelerated Repeated Rolling Wheel load Simulator, is proposed as an alternative to the current fatigue test technologies. The feasibility of the ARROWS is evaluated by comparing the stress state obtained from ARROWS with that in the multi-layered system.

## 2. Stress State in Asphalt Mixture under the Rolling Tire Load

The creation and accumulation of the fatigue damage in the asphalt mixture is caused by repeated rolling of tires on the surface of asphalt pavement. The good correlation between the fatigue results from the laboratory test methods and the field performance relies on how well (realistically) the repeated rolling loads have been simulated in the laboratory. Currently, it is the tensile stress at the bottom of the asphalt layer that attracts the most attention when designing asphalt pavement. Fatigue cracks are believed to be created at the bottom of the asphalt layer and propagate upwards. Given this, many studies employed cyclic one-dimensional tensile stress to evaluate the fatigue and cracking performance of the asphalt mixture. However, it is the two-dimensional tensile stress at the bottom of the asphalt layer that requires evaluation. Characterizing the tensile stress at the bottom of the asphalt layer under repeated rolling wheel loads is necessary to ensure the rationality of the fatigue test techniques.

**Figure 1:**
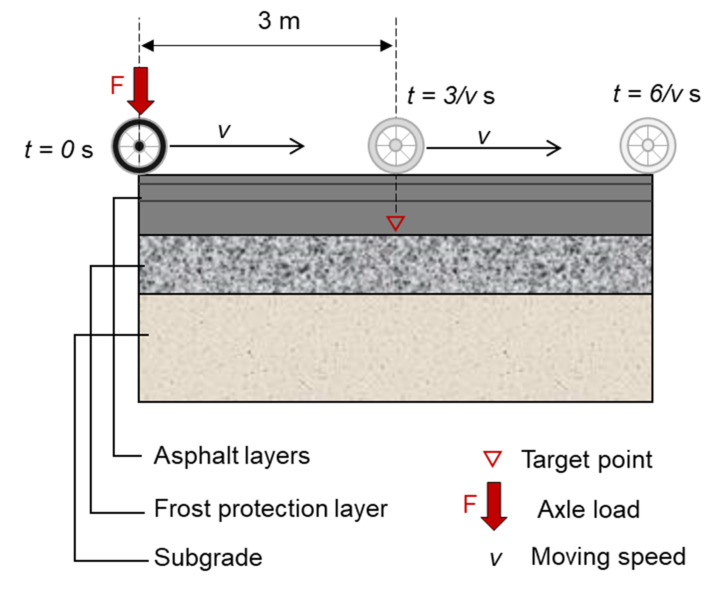
Schematic diagram of the pavement model.

**Figure 2:**
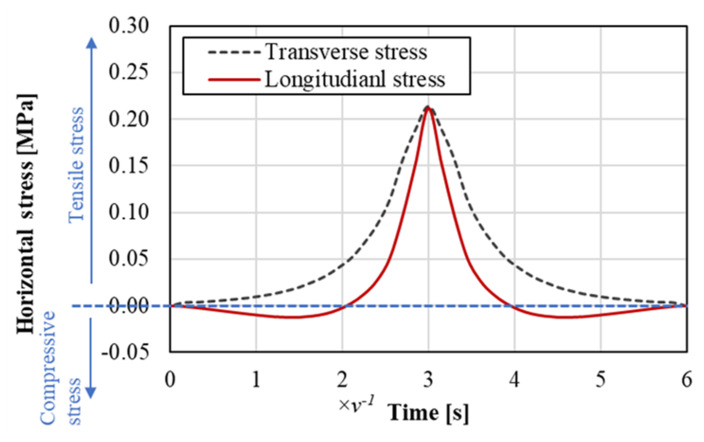
Stress reversal at the bottom of the asphalt layer.

The stress state in the asphalt mixture under repeated rolling wheel loads is analyzed in this section using the finite element (FE) method. In the FE model, a typical German pavement structure used for heavy traffic is simplified as an elastic multilayer system according to the standard RStO 12 [7]. The tire–pavement contact area and standard tire load are regarded as a circle with a diameter of 150 mm and 0.7 MPa, respectively, according to the standard RDO Asphalt 09 [8]. Table 1 details the information about pavement structure and involved materials. Figure 1 shows a schematic diagram of the pavement model. As shown, the wheel rolls towards the right side of the target point at a constant speed *v*. After *3/v* seconds, the wheel rolls to directly above the target point. The stress variation caused by the rolling process is shown in Figure 2.

Figure 2 presents the variation of horizontal stresses at the target point of asphalt mixture. As shown, both the transverse and longitudinal stresses reach the maximum when the wheel is directly above the target point. Transverse stress presents as pure tensile stress for the entire process. On the contrary, longitudinal stress experiences a compression–tension–compression process, which is called stress reversal in this paper.

An ideal fatigue test device is desired to reproduce the characteristics of tensile stresses on lab specimens. Currently, the lab test methods simplify the realistic stress waves as sinusoidal or haversine waves. By introducing a gap between two adjacent load waves, researchers can consider the self-healing performance while measuring the fatigue performance of the test specimen. However, Figure 2 indicates that the lab load on the specimen should be at least two-dimensional. Moreover, the stress at one dimension should contain a stress reversal and the stress at another dimension should be pure tensile stress.

## 3. Laboratory Fatigue Test Methods

The development of fatigue test devices was initially upon in the mid-1960s, where Professor Monismith et al. developed the UCB device, a 4PB fatigue test device [2]. After decades of development, there are a variety of fatigue test methods available. These methods vary from the fatigue test device and experiment setup to control methods. In general, there are two approaches to assess the fatigue properties of asphalt mixture. In the first approach, repeated loads are applied to test the specimen until the specimen fails. The responses of asphalt mixture are related to the number of loading repetitions. The second approach aims at measuring the material fundamental stress–strain relationship to formulate rigorous constitutive modes and evaluate the fatigue resistance properties of the corresponding asphalt mixture and pavement. The typical example of the second approach is the fracture mechanics-based critical strain energy release rate, Jc, derived from the notched semi-circular bending (SCB) test [9,10]. This paper primarily focuses on the test technologies employing the first approach. The involved fatigue test technologies are separated into bending fatigue test, axial fatigue test, diametral fatigue test, triaxle fatigue test, and shear fatigue test according to their loading modes.

### 3.1. Bending Beam Fatigue Test

#### 3.1.1. 4PB Fatigue Test

**Figure 3:**
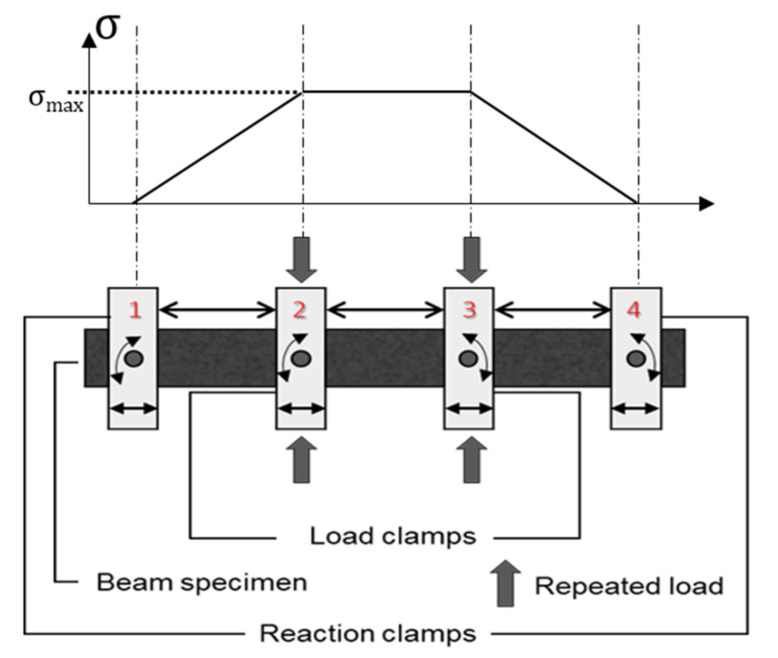
Scheme and tensile stress distribution of 4PB fatigue test.

The 4PB fatigue test is conducted on compacted beam specimens using a fixed reference point bending beam fixture. Figure 3 is the schematic diagram and the tensile stress distribution of the 4PB fatigue test. The beam specimen is fixed by two reaction clamps (clamps 1 and 4) on the outside. Repeated loads are introduced to the middle third of the specimen by two inside closed load clamps (clamps 2 and 3). The setups of the clamps allow the middle third of the prismatic specimen to work under maximum stress; the prismatic specimen is evenly distributed in the horizontal direction, and has zero moments. However, the flexural load in the specimen does not generate a homogenous stress distribution within the specimen.

In the 4PB fatigue test, the asphalt mixture specimen is subjected to repeated bending until it fails. The number of loading cycles at failure, i.e., fatigue life Nf, is recorded and plotted against the applied load value. The variation of flexural stiffness indicates the specimen’s response to repeated loading. The calculation of the stiffness modulus from the deflection measurement relies on the slender beam theory, in which deflection due to shear is ignored. The Saint-Venant’s principle derives the following formulas for calculating the maximum tensile stress and strain of the 4PB test where the loading span is 1/3 of the support span.

(1) $$\sigma_{t}=\frac{P\times L}{2\times b\times h^{2}}$$

(2) $$\varepsilon_{t}=\frac{54\times u\times h}{23\times b\times h^{2}}$$

(3) $$S=\frac{\sigma_{t}}{\varepsilon_{t}}$$

where

σ_t_ = Maximum tensile stress at the bottom of beam (Pa);
*P* = Peak-to-peak force applied on beam (N);
*b* = Average specimen breath (width) (mm);
*h* = Average specimen height (mm);
*L* = Length of beam span between outside clamps (mm);
ε_t_ = Maximum tensile strain at the bottom of beam (1);
*u* = Peak-to-peak deflection at center of beam (mm);
*S* = Flexural beam stiffness (Pa).

Similarly, by considering the plane hypothesis in elastic mechanics, Lv et al. proposed a methodology to calculate the compressive and tensile stress at the top and bottom of beam specimen. Using these formulas (Equations (4) and (5)), researchers are able to evaluate the compressive and tensile modulus evolution synchronously using 4PB fatigue test [11].

(4) $$E_{c}=\frac{P\times L\times \left(\varepsilon_{t}+\varepsilon_{c}\right)}{2\times b\times \varepsilon_{c}^{2}\times h^{2}}$$

(5) $$E_{t}=\frac{P\times L\times \left(\varepsilon_{t}+\varepsilon_{c}\right)}{2\times b\times \varepsilon_{t}^{2}\times h^{2}}$$

where

*E_t_* = Maximum tensile modulus (Pa);
*E_c_* = Maximum compressive modulus (Pa).

The 4PB fatigue test was first proposed by researchers from the University of California in Berkeley (UCB). Later, it was further refined during the SHRPA-003A project, completed in 1994, including the improvements to the data acquisition and control system and the use of sinusoidal loads [12]. Based on the research findings of this project, two standards, namely, ASTM D7460-08 and AASHTO T321-03, were developed. Over the years of research work, the ASTM D7460-10 and AASHTO T321-14 were released to unify the failure definition and achieve similar test results [13,14]. The main difference among these standards is the format of the repeated load waves. Because both ASTM and AASHTO methods are run in displacement-controlled mode, the format of load wave describes the waveform of the displacements imparted to the beam by the load clamps. The ASTM standard employs the cyclic haversine loads, in which the device bends the beam specimen on one side of its neutral axis [13]. The AASHTO standard recommends the use of the repeated sinusoidal loads, which means the applied displacement oscillates alternatively on both sides of the neutral axis with the same amplitude on both sides. Despite the AASHTO standard, the European standard EN 12697-24:2018 and the Australian standard AG: PT/T233-2016 suggest using cyclic sinusoidal loads as well [3,15]. Because the load waveform is not unified, cyclic loads with both sinusoidal and haversine waveform are utilized in the 4PB fatigue test. For instance, Yu et al., Dondi et al., Arsenie, et al., and Li et al. used the haversine displacement to assess the fatigue resistance of asphalt mixture [16,17,18,19,20]. On the other hand, Poulikakos et al., Di Benedetto et al., and Abhijith and Narayan employed sinusoidal loading in their studies [21,22,23]. Some concerns have been raised regarding the comparability of the 4PB fatigue test using different displacement waveforms. However, many studies reveal similar results of the 4PB fatigue test with same equivalent peak-to-peak strain in haversine and sine displacement testing modes [18,24]. A plausible explanation is due to the viscoelastic nature of asphalt mixture. Specifically, after a few loading cycles, asphalt mixture will exhibit a sinusoidal response to the applied haversine displacement, if the beam specimen is not supported by an elastic layer [18,25,26,27]. Given this, researchers believe it is not necessary to specifically recommend waveform of displacement in terms of the response of asphalt mixture. Nevertheless, this conclusion cannot be extrapolated to the asphalt mixtures whose nature is more elastic than viscoelastic. For instance, the strongly aged asphalt mixtures and the asphalt mixtures with harder asphalt binders. Besides, another limitation of these studies is the lack of consideration on the rest period. Mamlouk et al. induced a five-second rest period between two haversine load cycles [26]. Their findings reported the inconsistent results of 4PB fatigue test run in the haversine mode with and without rest period. Therefore, Mamlouk et al. recommended the ASTM to refine the ASTM D7460 by replacing the haversine waveform to sinusoidal waveform. It should be noted that in December 2018 the ASTM D8237-18 was published, in which the use of sinusoidal displacement waveform is recommended. Subsequently, the standard ASTM D7460-10 was withdrawn in 2019 [28].

Notably, the arguments regarding the waveform highlight the critical importance of load waveform to the fatigue response of asphalt mixture, which is also documented by the SHRP-A-003A report [29]. Besides, the rest period shows strong influences on the fatigue response of asphalt mixture by affecting the output force. Although many discussions have been conducted on the selection of load waveform, there are two consensuses: neither the sinusoidal nor the haversine waveform introduced by 4PB fatigue test realistically simulate the filed condition; and the elastic support foundation layer is important.

#### 3.1.2. 3PB Fatigue Test

**Figure 4:**
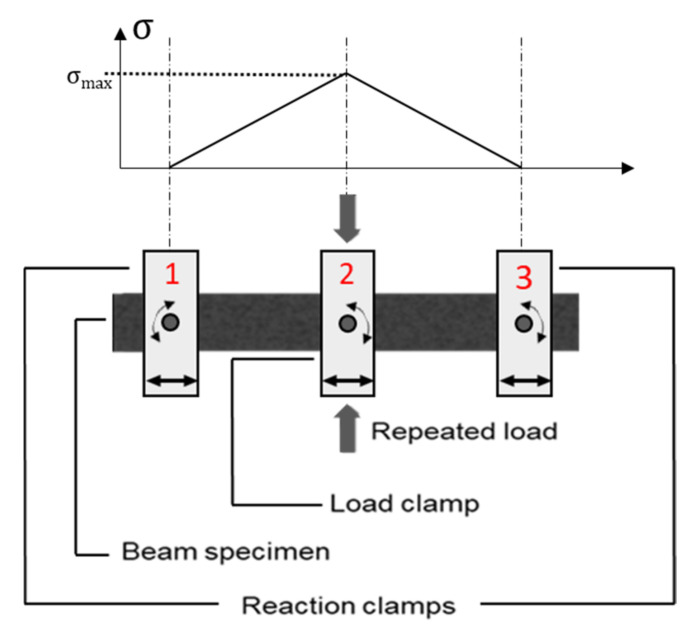
Schematic diagram and tensile stress distribution of 3PB fatigue test.

The 3PB fatigue test is also known as the center-point bending fatigue test [30]. This test is similar to the 4PB fatigue test, but the repeated loads are applied at the middle span of the specimen from a single point. Khalid believes the test duration of the 3PB fatigue test is likely to be reduced in comparison with 4PB fatigue test. This is because, for the same magnitude of applied force, the whole of the load applied by the load cell in 3PB test is transferred to the sample, whereas in the 4PB test, the applied load is split in two. However, because the tensile stress is exactly maximum under the point load, 3PB test does not allow the initiation of failure in a relatively uniform tensile stress region [29]. The scheme of the 3PB test and the distribution of the tensile stress is shown in Figure 4.

The maximum tensile stress of the 3PB test is mathematically expressed as below:

(6) $$\sigma_{t}=\frac{3\times P\times L}{4\times b\times h^{2}}$$

#### 3.1.3. 2PB Fatigue Test

2PB fatigue test, which was first developed in France, is widespread in European countries as a standard fatigue resistance test method [3]. This test is performed on a trapezoidal or prismatic beam set up in a test machine as a cantilever beam. The 2PB fatigue test is desired to be suitable for all types of asphalt mixture with the geometry of the sample as a function of the maximum nominal aggregate size (MNAS) of the asphalt mixture. The main result of the 2PB fatigue test is further used for pavement design. Specifically, the French pavement design principle utilizes the strain ε6, which is defined as the strain for failure at one million load repetitions using the 2PB fatigue test on the trapezoidal specimen, as the only metric of fatigue resistance.

**Figure 5:**
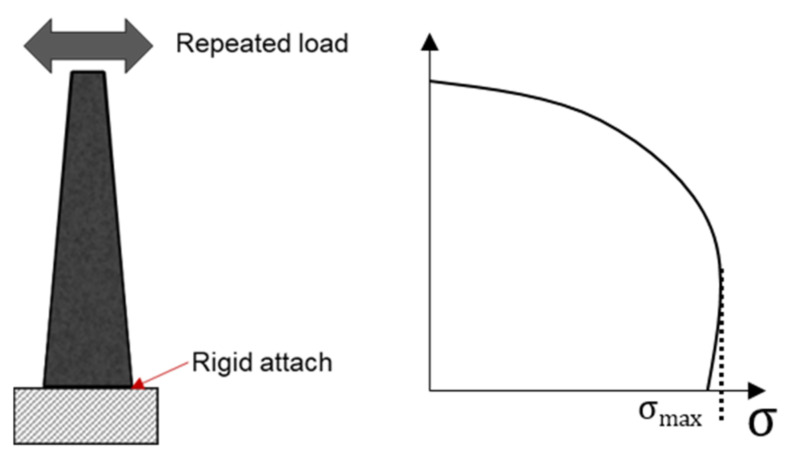
Schematic diagram and tensile stress distribution of 2PB fatigue test.

During the 2PB fatigue test, the sample is installed vertically, its wide end is rigidly attached to a metal support, while its narrow end is subjected to repeated horizontal loads. Figure 5 shows a 2PB fatigue test device with a trapezoidal sample and the associated tensile stress distribution.

The applied loads are sinusoidal only but can be in either stress-controlled or strain-controlled mode [23,31]. The force–displacement used to generate the loads is recorded and used to identify fatigue failure of the sample. The specimens will fail at about mid height where the flexural stress occurs. The correlation between the flexural strain level and the displacement applied to the specimen head (narrow end of the specimen) can be described by a constant depending on the specimen geometry [3].

(7) $$\varepsilon =K_{\varepsilon }\times z$$

(8) $$K_{\varepsilon i}=\frac{{\left(B_{i}-b_{i}\right)}^{2}}{8\times b_{i}\times h_{i}^{2}\left[\frac{\left(b_{i}-B_{i}\right)\times \left(3B_{i}-b_{i}\right)}{2\times B_{i}^{2}}+ln\frac{B_{i}}{b_{i}}\right]}$$

where

*ε* = Relative strain of the specimen (-);
*K_εi_* = Constant in relation to the largest strain;
*z* = Amplitude of the displacement at the head of the specimen;
*B_i_* = Length of long base edge;
*b_i_* = Length of short base edge;
*h_i_* = Height of the specimen (mm).

The main advantages of the 2PB fatigue test are its relatively simple experimental setup compared to other fatigue tests, and the ability to include two samples simultaneously. Several duplicate tests are required to precisely determine the Nf of asphalt mixture at a load level because the scatter of the fatigue test results is somehow inevitable. The 2PB fatigue test reduces the time needed to test the required set of samples as the 2PB fatigue test allows one to produce tests on two samples at the same time, indicating the same test condition and less scatter. As with any forms of bending fatigue tests, the weakness of the 2PB fatigue is the inhomogeneous stress distribution within the sample. In addition, Dondi et al. questioned the effects of sample gravity force on the 2PB test results. The logic behind this concern is that the direction of repeated loads employed by both 4PB and 3PB are parallel to the specimen’s gravity force, while that of 2PB is perpendicular to the gravity. Consequently, they revised the classical 2PB fatigue test to a horizontal 2PB fatigue test. In the horizontal 2PB fatigue test, repeated loads are applied to the horizontally installed test specimen [24]. It should be noted that the fabrication of the high quality and precise trapezoidal sample constitutes an important challenge in this test.

Because both the 2PB and 4PB fatigue tests are the standard European test methods, their results are desired to show close agreement. However, the fatigue test results are expected to be considerably affected by test type and the mode of loading [23]. Therefore, many studies have been performed to determine the differences in the results measured by 2PB and 4PB methods. Poulikakos et al. compared the complex modulus and fatigue performance of filed aged specimens using 2PB and 4PB tests. Their study revealed that the 2PB and 4PB tests show a good linear regression for complex modulus values but dissimilar fatigue performance ranking [22]. Di Benedetto et al. reported similar results. They found the complex modulus is independent of the test method, but the fatigue performance is considerably affected by the test type [23,32]. Pronk et al. found the fatigue life obtained by 2PB test was shorter compared to that measured by 4PB test [25].

#### 3.1.4. Loaded Wheel Fatigue Test

The loaded wheel fatigue test is derived from the wheel tracking tester, which is commonly used for evaluating the rutting resistance and moisture susceptibility of asphalt mixture. The loaded wheel fatigue test is desired to simulate the effects of a rolling wheel on the pavement by applying repeated moving wheel loads to the specimen [33,34]. To simulate the supporting effects of the base layer, in some studies, the specimen is placed on an elastic mat [35,36].

**Figure 6:**
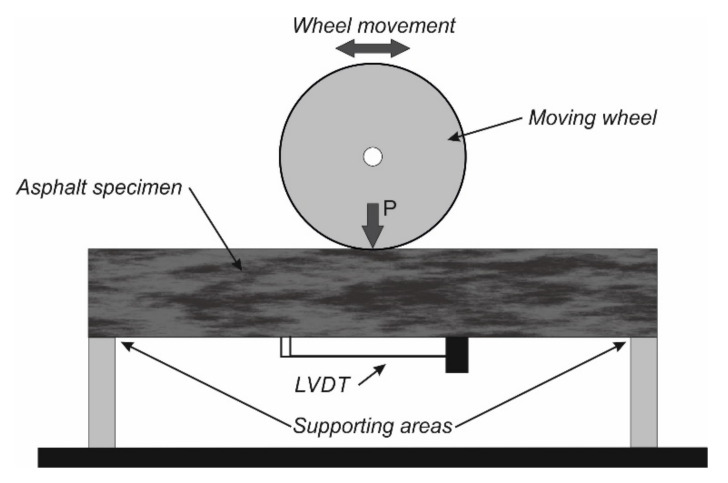
Scheme of the LWT fatigue test setup according to [38].

Wu et al. modified the asphalt pavement analyzer (APA) to perform an asphalt fatigue test (Figure 6) [37,38]. In their study, a beam specimen is subjected to repeated moving loads without supporting mat. A linear variable differential transformer (LVDT) was mounted on the bottom surface of the beam specimen to accurately measure the tensile strain under the moving load. To determine the tensile stress at the bottom surface of the specimen, the mechanical model of the test system was simplified as a plane-stress problem, within which, a vertical load moves on a beam.

Combining this simplification with the 2D slender beam theory derives the tensile stress at the midpoint of the beam bottom:

(9) $$\sigma_{t}=\sigma_{amp}\sin^{2}\left(\frac{2\pi }{T}t\right)=\frac{3PL}{2bh^{2}}\sin^{2}\left(\frac{2\pi }{T}t\right)$$

where

*σ_amp_* = Amplitude of sinusoidal stress (Pa);
*T* = Testing cycle period (s);
*t* = Elapsed testing time (s);
*P* = Wheel load (N);
*L* = Length of the loading path (m).

Equations (10) and (11) yields the dynamic modulus (E*) and phase angle (*δ*) of test specimen:

(10) $$E^{*}=\frac{3PL}{2bh^{2}\varepsilon_{amp}}$$

(11) δ=2πfΔt

where

*ε_amp_* = Amplitude of sinusoidal strain (-);
*f* = Loading frequency (Hz);
∆*t* = Time lag between stress and strain (s).

Wu et al. evaluated the fatigue resistance of four types of asphalt mixture using modified APA test, direct tensile test, and 4PB test. The modified APA test gave the same ranking of the four asphalt mixtures in terms of fatigue resistance; the 4PB test indicated the feasibility of using loaded wheel fatigue test to differentiate between asphalt mixtures in terms of their fatigue performance [38].

However, the unsupported LWT fatigue test brings obvious vertical deformation to the test specimen, which raises concerns about the capability of the unsupported LWT fatigue test to simulate the fatigue failure process of real pavement structure [36]. Alternatively, Zhang et al. investigated the fatigue behavior of asphalt mixture using the modified Hamburg Wheel-Tracking Device (HWTD) [36]. Compared with APA modified by Wu et al., the main differences of the modified HWTD are the supporting condition and the side-confining condition. Zhang et al. utilized two 20-mm thick neoprene layers to simulate the supporting effects of the pavement base layer. The neoprene was selected as its modulus is similar to that of the pavement base course. The prismatic asphalt mixture sample was horizontally sandwiched by two wood strips, which was also used to simulate the side-confining environment. To monitor the response of the test specimen to cyclic loads, strain gauges were attached under the test specimen. Obviously, the modified HWTD system is a complex three-dimensional system, indicating the inapplicability of the slender beam system in calculating the bottom tensile stress. In their study, tensile stress is assumed as constant during the cyclic loading process. Based on this assumption, the fatigue response of asphalt mixture was analyzed using energy method.

**Figure 7:**
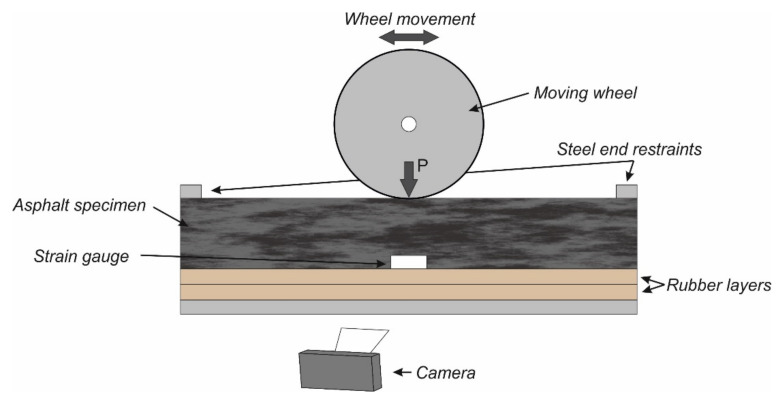
Scheme of experimental setup of BWTFT according to [39].

Similarly, Nguyen and Thom characterized the fatigue performance of asphalt mixture using a Beam Wheel Tracker Fatigue Test (BWTFT) [39]. The BWTFT was developed based on the wheel tracker machine from the Nottingham Transportation Engineering Center (NTEC). In their study, two 10 mm-thick rubber mats were used to beneath the asphalt beam. Cyclic loads were applied to the beam specimen through the loaded wheel. Strain gauges were attached on the two sides of the beam, as shown in Figure 7, to monitor the response of the asphalt beam to moving wheel loads. The peak–trough strain collected using the strain gauges was plotted against the number of loading cycles as the output result of the BWTFT test.

Nguyen and Thom treated the BWTFT as a beam on an elastic foundation layer. The supporting effect of the rubber mat is a function of rubber modulus and curvature, which derives the tensile stress at the midpoint of the asphalt beam:

(12) $$\sigma_{t}=\frac{1.14\times E^{0.25}\times P}{k^{0.25}\times h^{1.25}}$$

where

*E* = Modulus of asphalt (Pa);
*k* = Modulus of rubber (Pa);
*h* = Beam thickness (m).

The GoPro camera was used to capture the cracking behavior of asphalt mixture. Apart from the cracking behavior, the collected sample photos also indicated that no obvious vertical deformation occurs on the failed specimen, which is ascribed to the supporting effect of the rubber mat.

The main shortcoming of the loaded wheel fatigue test is its loading frequency. Due to the limitation of the LWT device, the loading frequency is usually low and nonadjustable. Specifically, the loading frequency used by Wu et al., Zhang et al., and Nguyen and Thom are 2 Hz, 1 Hz, and 0.1 Hz, respectively [36,38,39].

### 3.2. Axial Fatigue Test

The axial fatigue test refers to the test methods that apply uniaxial cyclic loads to specimen with or without stress reversal. The mechanism of the axial fatigue test brings two advantages compared with other technologies: the homogeneous distribution of applied load and ease of control and measurement. Specifically, in the axial fatigue test the load and deformation are distributed regularly over the cross-section of the specimen under loading. The axial fatigue test allows pavement engineers to directly collect the responses of test specimen in the form of force–displacement without any further process. Because of these merits, axial fatigue test plays a critical role in evaluating the fatigue performance of asphalt mixture and the development of damage theories of asphalt mixture [6,40,41,42,43,44].

Typical test methods of the axial fatigue test include the direct tension fatigue test (DTFT), the compression fatigue test (DCFT), and the tension compression fatigue test (TCFT). During these tests, loads are applied to the top end of the sample through the actuator. As with other test technologies, the applied cyclic loads of axial fatigue test can be controlled in force or displacement mode [45,46].

For the test methods involving uniaxial tension load, an encountered experimental problem is the end-failure of the specimen, which refers to the fatigue failure at the ends of the specimen. There are two possible reasons for the end-failure phenomenon: non-uniformity distribution of the air voids and the eccentric load [45,47]. The ends of cylinder specimen fabricated by Superpave gyratory compactor (SGC) exhibits higher air void content, increasing the likelihood of failure at the corresponding points [48,49,50]. Eccentric tension is believed to be another critical factor leading to the premature end-failure [51]. The maximum axial stress and corresponding strain at the sample end increase sharply with the increase in load eccentricity. The proper experimental setup is necessary for avoiding eccentric tension [52,53]. Therefore, both TCFT and DTFT require test samples to have a firm connection to the test system. Enough adhesive bond between the sample ends and the test system is necessary to avoid the adhesive failure at the sample–glue or glue–platen interface under tension loading.

CFT is relatively less used in evaluating the fatigue resistance of asphalt mixture, which may be because compression load is more related to the permanent deformation, such as rutting at high-temperatures [54,55,56]. However, CFT could contribute to formulating fatigue damage models for asphalt mixture. For instance, Lv et al. jointly used the CFT, DTFT, and indirect tensile fatigue test (ITFT) methods to build a normalized model of fatigue characteristics for asphalt mixture in three-dimensional stress states [57].

DTFT method employs cyclic uniaxial tension loads to determine the fatigue response of asphalt mixture, the waveform of cyclic loads can be in either sinusoidal or haversine waveform mode [58,59]. A standard test protocol (AASHTO TP107) and analytical method have been developed based on the DTFT setup [60,61,62]. As with other test technologies, DTFT was also able to investigate the fatigue response of asphalt mixture, considering the many variables, such as loading frequency, rest period, material properties, aging condition, and test temperature [63,64,65,66].

**Figure 8:**
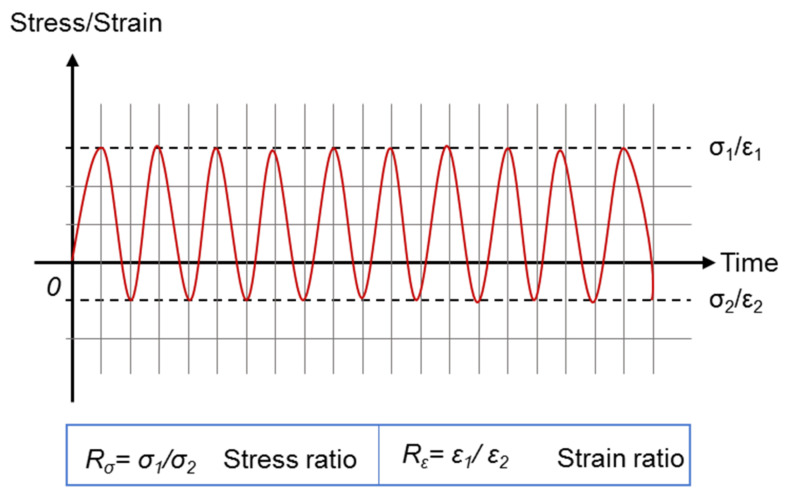
Definition of stress–strain ratio.

TCFT, as expected, contains a stress reversal for each loading cycle, because of which, TCFT can simulate the stress reversal observed in the field condition. Both strain-controlled mode and stress-controlled mode apply to the TCFT [46,67,68]. Regarding the load waveform, only the sinusoidal waveform is reported by the surveyed literature. In comparison with DTFT, TCFT offers the possibility to consider the effects of stress–strain ratio R, which is defined by Figure 8. As reported by Isailović et al., introducing a stress reversal into the loading waveform reduces the energy dissipated and partially eliminates the vertical deformation of the test sample [46].

Later, Zhang and Oeser derived a damage evolution model from the continuum damage mechanics [6]. According to their model (Equation (13)), *N_f_* is a function of material parameters *α* and *β*, and peak-to-peak stress *σ*:

(13) $$N_{f}=\frac{\alpha }{\left(1+\alpha \right)\beta }\sigma^{-\alpha }$$

where α > 1.

Replacing σ by $\sigma =\sigma \left(\sigma_{1},R\right)$ yields:

(14) $$N_{f}=\frac{\alpha }{\left(1+\alpha \right)\beta }\cdot {\left(\frac{R_{\sigma }-1}{R}\right)}^{\alpha }\cdot {\left(\frac{1}{\sigma_{1}}\right)}^{\alpha }$$

Equation (12) indicates that *N_f_* depends on the material parameters, stress ratio, and *σ*_1_. For example, if the *σ_1_* remains the same and *R_σ_* changes from −1 to −2, *N_f_* will increase by (4/3)α times. Further studies are demanded to comprehensively investigate the effects of *R* on the fatigue performance of asphalt mixture.

**Figure 9:**
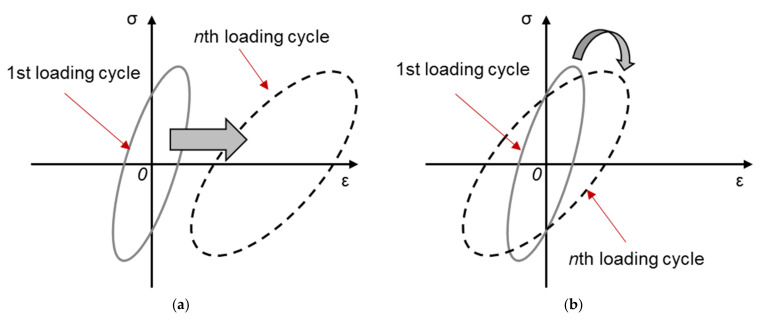
Changes in form and position of hysteresis loop. (**a**) Horizontal moving. (**b**) Rotation and expansion.

The primary limitation of the DTFT is that the utilized pure tension loads do not necessarily represent the field conditions. In addition, DTFT under stress-controlled mode is criticized because it creates permanent vertical deformation to the specimen [46]. According to Di Benedetto, asphalt mixture exhibits two kinds of material behavior under the cyclic loading: accumulation of permanent deformation and the aggravation of fatigue damage [23,69]. These two material behaviors can be separated by the form and position of the hysteresis loop in each loading cycle, as shown in Figure 9. Rotation and expansion of the hysteresis loops with increasing number of loading cycles are primarily due to material fatigue, while the horizontal moving of the loops is ascribed to the permanent deformation [69].

Isailović et al. observed the horizontal moving of the hysteresis loops of asphalt mixture under uniaxial tension stress, indicating that the failure of asphalt mixture under uniaxial tension stress is primarily associated to the accumulation of permanent deformation. Hence, they do not recommend using DTFT for fatigue analysis [46]. Similarly, in comparison with DTFT, Ashouri recommended the use of zero-mean stress-controlled TCFT instead of the zero-minimum stress-controlled DTFT to evaluate the healing behavior of asphalt mixture due to the following considerations [70]: first, TCFT can better simulate the pavement stress history than the DTFT; second, zero-mean stress-controlled TCFT makes the comparison between the fatigue test without rest periods and the healing test with reset periods easier, and third, fatigue failure is defined as the cycle when the phase angle starts to decrease. This trend is only found in the results of TCFT and not in the results of DTFT. A plausible explanation of this phenomenon is that the accumulation of viscoplastic strain prevents the material to heal.

Overall, the axial fatigue test technologies purely focus on the material property regarding fatigue performance. The loading conditions of these technologies do not necessarily represent the field conditions caused by the horizontal moving of wheel tires. Although introducing stress reversal into the loading waveform can simulate the loading pulse caused by the passing of wheel loads on pavement, TCFT does not consider the structural effect of pavement and the realistic stress–strain ratio.

### 3.3. Diametral Fatigue Test

Diametral fatigue tests refers to the tests that apply cyclic loads to the specimen in the diameter direction of the specimen. A typical diametral fatigue test is the indirect tension fatigue test (ITFT).

ITFT is one of the four fatigue test technologies recommended by the European standard [3]. Pavement engineers are able to design asphalt mixtures and pavements for fatigue adequacy based on the ITFT-measured fatigue response together and its correlation with the field performance.

**Figure 10:**
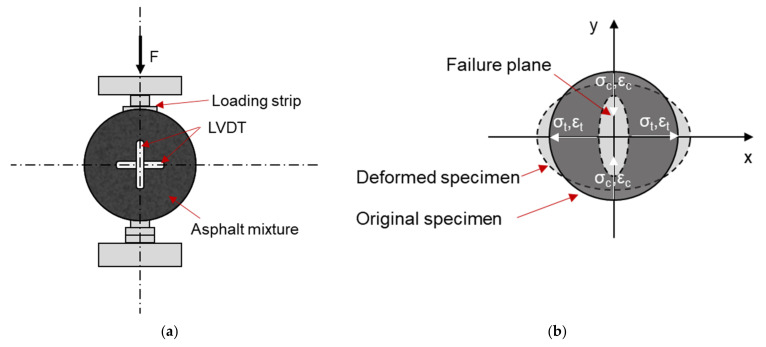

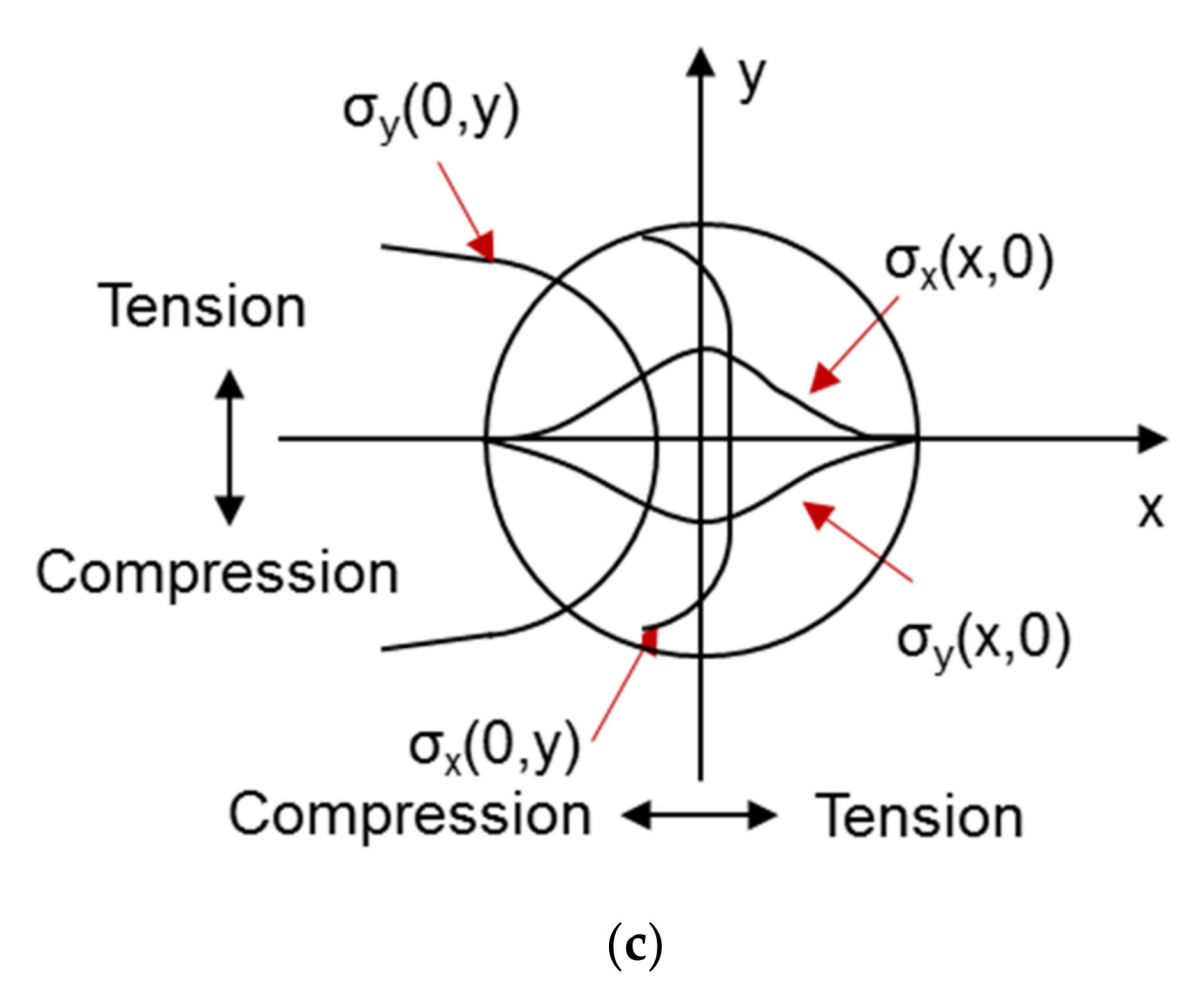
Schematic diagram and stress distribution of ITFT. (**a**) ITFT test setup. (**b**) Stress and strain on the diameters. (**c**) Stress distribution along horizontal and vertical diameters.

As shown in Figure 10a, repeated vertical loads are imposed on the cylinder samples. Figure 10b illustrates that the loading configuration develops a reasonable uniform tensile stress in the specimen perpendicular to the direction of the applied load and along the vertical diametral plane. Figure 10c shows the stress distribution in the specimen on the diameter. During the ITFT, both the cyclic haversine and cyclic sinusoidal loads are employed [3,6,23,71,72,73]. The loading strip is employed to prevent the specimen from failing near the load line due to compression [29].

Under this configuration, the compressive and tensile stresses at the center of specimen are as follows [74]:

(15) $$\sigma_{t}=\frac{2\times P}{\pi \times a\times h}\times \sin\left(2a-\frac{a}{2R}\right)$$

(16) $$\sigma_{c}=\frac{-6\times P}{\pi \times a\times h}\times \sin\left(2a-\frac{a}{2R}\right)$$

where

*σ_t_* = Horizontal tensile stress at the center of specimen (Pa);
*σ_c_* = Vertical compressive stress at the center of specimen (Pa);
*a* = Width of loading strip (m);
*h* = Height of specimen (m).

Equations (15) and (16) yield that at the center of specimen, the vertical compressive stress is three times the horizontal tensile stress.

As shown in Figure 10b, the biaxial state of stress induced by ITFT is the main difference between the ITFT and the other technologies mentioned above. In the biaxial stress state, the maximum stress value is concentrated in the center of the specimen and declines outside. The biaxial stress is possibly of a type better representing the filed condition. However, because the ratio of vertical compressive stress to horizontal stress is fixed at three, the goodness of ITFT in representing the field stress state is questioned. Besides that, the current specification recommends the use of horizontal stress and strain to evaluate the fatigue performance of asphalt mixture. A plausible approach to consider the influences of the biaxial stress state is still missed. Moreover, the haversine pattern of loading raises two concerns about the ITFT technology. First, if σt is used to evaluate the fatigue response of asphalt mixture, the absence of stress reveal may compromise the correlation between experimental results and field fatigue performance. Second, this loading pattern creates a permanent deformation under the loading strips, especially at an elevated temperature [75,76]. In this case, the ITFT causes a combination of damage from fatigue and permanent deformations [31].

A number of studies have revealed that ITFT does not deliver the same results with the other test methods in terms of fatigue performance of asphalt mixture. For instance, Di Benedetto et al. reported that ITFT provides shorter fatigue lives when compared with bending tests [23]. Poulikakos and Hofko compared the fatigue lives of asphalt mixture measured by ITFT and 4PB [77]. According to them, ITFT and 4PB did not provide the same results, both in terms of value and ranking. Cheng et al. applied strain-controlled and stress-controlled cyclic haversine loads to 4PB and ITFT specimens, respectively, at different temperatures. They found that temperature shows positive influences on the Nf measured by 4PB but negative influences on the Nf measured by ITFT [78]. Yu et al. ranked the fatigue performance of asphalt binder, asphalt mortar and asphalt mixture using different test methods. They found the results of ITFT were not consistent with the results of the other methods [71].

### 3.4. Shear Fatigue Test

**Figure 11:**
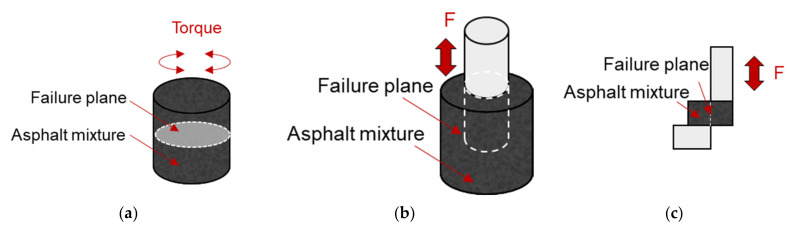
Schematic of shear fatigue test configurations. (**a**) Torsional shear. (**b**) Penetration shear. (**c**) Direct shear.

At present, several test methods are available to characterize the shear fatigue performance of asphalt mixture, and these methods can be categorized as two types: torsional and direct shear fatigue test method. Ragni et al. applied shear-torque configuration to characterize the interlayer fatigue performance of double-layered asphalt mixture specimen [79]. Ren et al. designed a uniaxial penetrating test (UPT) method to characterize the shear fatigue performance of asphalt mixture [80]. Yin et al. [81] and Rahman et al. [82] evaluated the fracture behavior of asphalt mixture under direct shear loading. The schematics of the torque- and direct-shear configurations are shown in Figure 11.

However, it should be noted that compared with fatigue distresses, the shear resistance of asphalt mixture is considered to be more related to the rutting of asphalt mixture [83]. This is because shear flow of asphalt mixture is ascribed as one of the major reasons leading to rutting [84].

## 4. Full Scale Test Method

Full-scale test method lies on the full-scale pavement structure, which is loaded by the repeated axle loads or the accelerated axle load. Because of the involved full-scale pavement structure and the realistic axle loads, the full-scale method is regarded as a reliable method to better understand and characterize the performance degradation process of asphalt pavement. The experimental data collected from these methods are widely used to verify the damage evolution models in the pavement design guide.

The typical full-scale test facilities include the AASHO road (Ottawa, Illinois, USA) [85], WesTrack (Reno, Nevada, USA) [86], NCAT test track (Opelika, Alabama, USA) [87], MnROAD (Otsego, Minnesota, USA) [88], and RIOHTrack (Beijing, China) [89]. Apart from the full-scale test tracks, hydro-servo/weight loading devices are also available for evaluating the fatigue performance of pavement. In this method, repeated loads are introduced to the full-scale or model-scale pavement in a linear or circular track. The typical ring tracks are the CEDEX (Madrid, Spain) [90], CAPTIF (Christchurch, Canterbury, New Zealand) [91], LCPC (Nantes, Pays de la Loire, France) [92], ALF (Australia) [93], HVS (South Africa) [94], and MLS (United Kingdom) [95].

With the assistance of full-scale test methods, pavement researchers are able to determine the effects of traffic loads on the performance degradation of pavement structure. The advantage of these test tracks is that they can represent the real service condition of the pavement structure. However, the shortcoming is the high cost of the experiment and the large area. In addition, there is still a gap between the test loading frequency and the real traffic speed.

## 5. Summary and Discussion

After decades of effort, plenty of test technologies have been proposed to investigate the fatigue behavior of asphalt mixture. Although the sample geometry and load characteristics involved in these technologies vary from method to method, these technologies are the base stone of understanding and predicting the fatigue behavior of asphalt materials on different length scales. Table 2 provides an overview of the test methods and their parameters by showing their loading types, the measured properties, failure zone description, stress characteristic, and stress reversal.

Despite their contributions, comparing the field service conditions with the test conditions highlights the limitations of the currently used technology: the lack of considering the influences of pavement structure and the characteristics of traffic load.

With laboratory test methods, most of them focus on the fatigue behavior of test material only without considering the influences of pavement structure. Previous studies have documented that the structural configuration can sometimes overshadow the effect of mixture property on fatigue performance, delivering different results between the laboratory fatigue performance and field fatigue performance [96,97]. During each loading cycle, the multi-layer characteristic of asphalt pavement structure resulted in a stress reversal at the longitudinal direction in the asphalt mixture, but not at the transverse direction. Stress reversal affects the fatigue performance of asphalt mixture by varying the stress ratio, however, neither the stress ratio nor the two-dimensional stress state was well simulated in the lab test methods. To omit the negative influences of the lab methods, several empirical parameters, which are determined statistically, are required to link the lab measured results to the field condition. In addition, the utilized diverse loading methods induce the non-unifying nature regarding ranking fatigue performance. When ranking the fatigue performance for a group of samples, different methods may yield different ranking results, which causes difficulty in selecting the optimal paving materials. The full-scale test methods appear to be the solution for the limitation of the lab methods. However, the high cost of the test facilities obstacles in their application.

A common shortcoming of both the lab methods and the full-scale methods is the loading speed. The importance of loading speed on the fatigue performance of asphalt materials has been reported by numerous studies. Due to their technical limitations, the maximum loading speed (approx. 20 km/h) of the hydro-servo/weight loading test devices is much slower than the real traffic speed [4,98,99,100], resulting in a significant difference between the test and the real service condition. The lab devices can apply load at a relatively high frequency. However, the loading frequency varies among the test standards. The ASTM D8237-18 recommends a default frequency of 10 Hz for the 4PB fatigue test [28]. The AASHTO standard requires a frequency range of 5 Hz to 25 Hz for the 4PB fatigue test [14]. The frequency for the 2PB fatigue test, however, is fixed at 25 Hz [3]. A plausible explanation is the relationship between the loading frequency and traffic speed, though this remains unclear. Table 3 lists the formulas used to converse the traffic speed v (km/h) to the lab loading frequency.

## 6. Concept and Development of ARROWS

### 6.1. Characteristics of ARROWS

Previous sections have shown the differences in the loading condition between the existing fatigue test methods and the field. These differences lead to a gap between the fatigue performance of asphalt mixture in the lab and in the field. A plausible solution for the insufficient correlation is to develop a new fatigue test device, which is able to reproduce the field loading conditions in lab. To achieve this purpose, the authors propose the concept of an innovative fatigue test device, which is called Accelerated Repeated Rolling Wheel Load Simulator (ARROWS). Because the device is under construction, this section briefly introduces the concept and mechanism of the device; practical work will be performed once the device is available.

To better reproduce the field service conditions, ARROWS is expected to have the following technical characteristics: (a) ARROWS can generate two-dimensional tensile stress at the bottom of asphalt mixture layer, (b) the loading speed should be comparable to the traffic speed, and (c) each loading pulse should consist of a loading phase and a rest phase.

**Figure 12:**
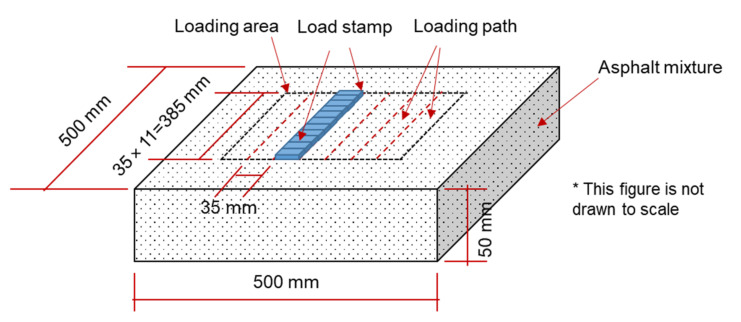
Geometry of asphalt mixture slab.

The ARROWS employs a rectangle asphalt mixture slab with the dimension of 500 mm × 500 mm × 50 mm (length × width × thickness), as shown in Figure 12. A rubber mat is used to simulate the supporting effect of the base layer on the asphalt mixture, which has been documented by previous studies. The thickness of the asphalt slab and the rubber mat is adjustable to simulate different pavement structures. During the test, repeated loads are imposed on the loading paths through eleven load stamps. The contact area between the load stamps and the asphalt mixture is set as 35 mm × 35 mm and can be adjusted upon request.

As mentioned in the above sections, the wheel moving speed of the APA system is much slower than the traffic speed. In order to reproduce the moving process of wheel load at traffic speed in the lab, the ARROWS involves a different loading method. Instead of applying load to the sample using a moving wheel, ARROWS applies the moving load by changing the load magnitude of stamps with time. Specifically, if the load magnitudes of all the eleven stamps at instant t can be described mathematically, for instance, the Gaussian distribution (Equation (17)).

(17) $$F_{\mu ,\sigma^{2}}\left(n\right)=\frac{1}{\sigma \sqrt{2\pi }}e^{-\frac{1}{2}{\left(\frac{n-\mu }{\sigma }\right)}^{2}}$$

where parameter *μ* is the expectation of the distribution, the parameter *σ* is the standard deviation, and *n* is the number of the load stamp.

**Figure 13:**
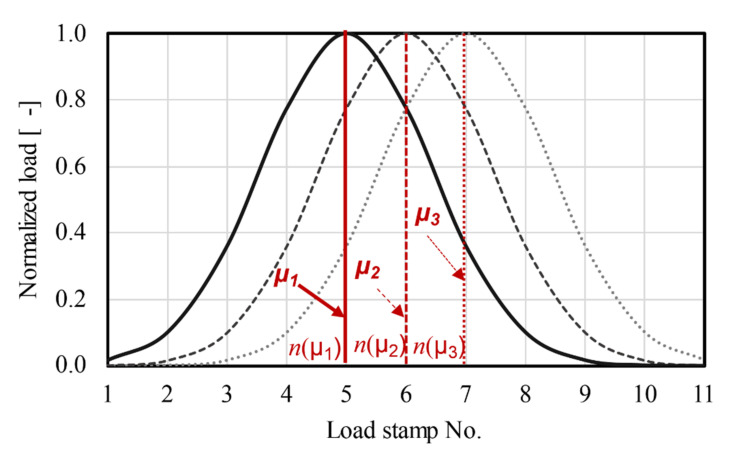
Load profile of stamps with different *μ* values.

The load profile of stamps at t(μ1), t(μ2), and t(μ3) are visualized as Figure 13. From this figure, it can be found that the load peak moves among the load stamps with time, which indicates that the repeated rolling of tire loads can be reproduced by changing the load profile with time t, and the corresponding loading speed v can be represented as:

(18) $$v=\frac{d*\left(n\left(\mu_{2}\right)-n\left(\mu_{1}\right)\right)}{t\left(\mu_{2}\right)-t\left(\mu_{1}\right)}$$

where *d* is the width of the load stamp.

### 6.2. Description of ARROWS

**Figure 14:**
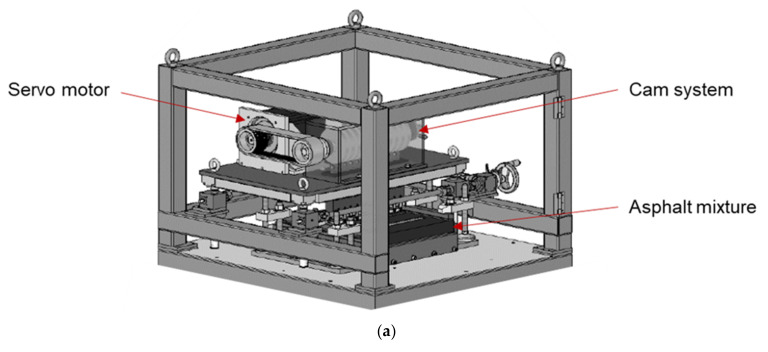

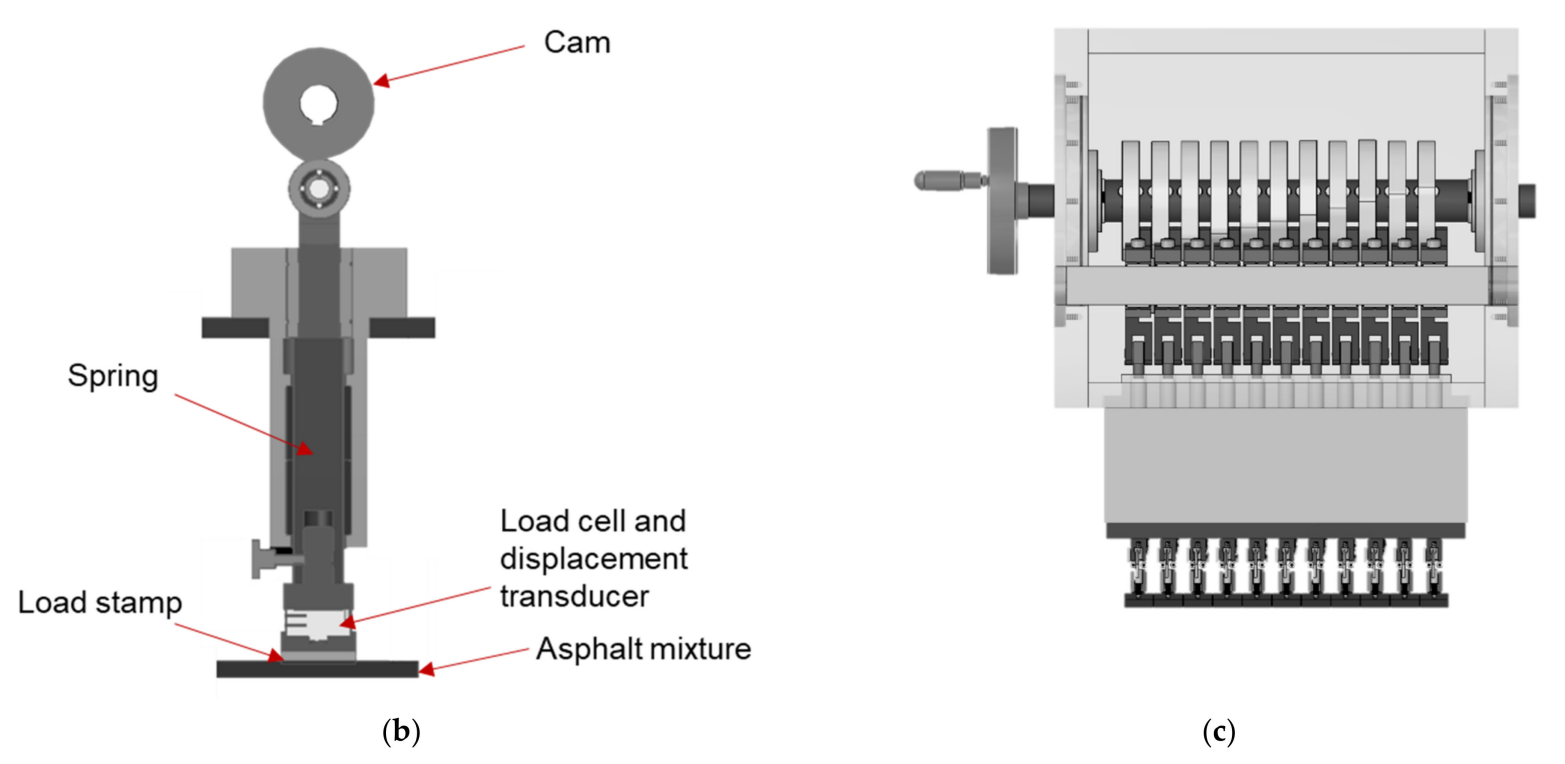
Details on the ARROWS. (**a**) Schematic diagram of ARROWS. (**b**) Side view of a loading unit. (**c**) Front view of the loading system.

Stamps is used to the create load on the asphalt mixture sample. In order to reduce the necessary preload of the system, which is required to ensure contact between the load stamp contact surface and the specimen, a wheel located in the cam plate is used as a counter bearing. The setup of the counter bearing is illustrated in Figure 14b. As seen, the load stamp is connected to the cam through a spring and a piston. Vertical displacement is induced to the top of the spring, when cam contacts with the piston. The compressed spring then transfers the load to the stamp. Hence, the magnitude and waveform of the applied load are determined by the geometry of the cams.

The measurement system of the ARROWS includes the load cells, the displacement transducers, and the strain gauges. Each of the eleven load stamps is equipped with a load cell and a displacement transducer, which are operated and sampled absolutely synchronously. Figure 14b reveals the setup of the load cell and displacement transducer. The load cells and the displacement transducers are used to collect the applied vertical load and the vertical displacement of the sample, respectively. Strain gauges are attached to the selected positions on the bottom of the asphalt specimen to determine the strain evolution at the bottom of the sample.

The eleven employed cams are arranged on the shaft (Figure 14c) with an adjustable angle between them. The geometry of the cam plates and their arrangement on the shaft are strongly coupled with the loading speed, which can be controlled by the rotational speed of the shaft.

**Figure 15:**
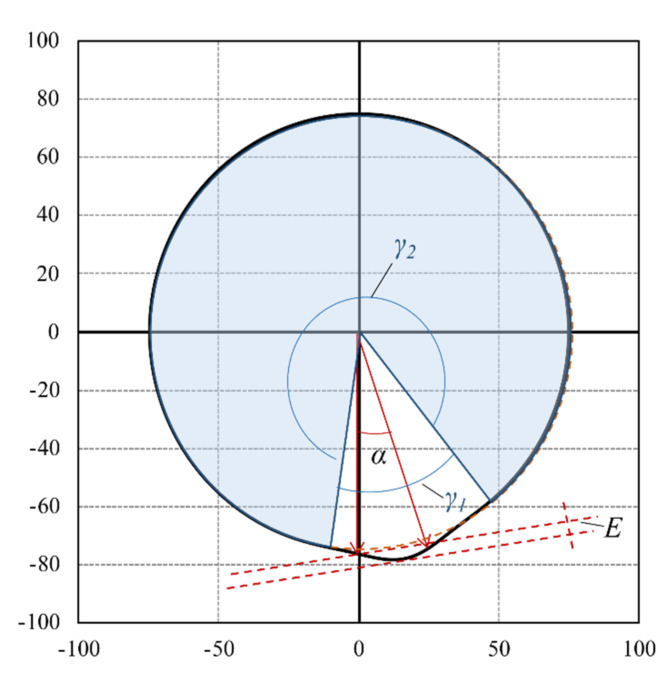
Schematic diagram of a cam plate.

Apart from reproducing the rolling motion of a wheel, ARROWS is designed to be capable of ensuring that the introduced work remains constant, regardless of the positions of the individual cams. This requirement is met by a defined geometry of the cam discs, which is described using a Gaussian function (Equation (17)). A parameter b is introduced to describe the ratio of *γ*_1_ to *γ*_2_. The physical meaning of parameter b is the ratio of the duration of the loading phase to that of the rest phase of each loading pulse. Figure 15 is the schematic diagram of a cam plate.

(19) $$r\left(\alpha \right)=E\cdot e^{f\cdot {\left(\frac{\alpha }{180{}^{\circ}}-1\right)}^{2}}+\frac{d}{2}$$

(20) $$f=\frac{d^{2}\cdot \pi^{2}\cdot b^{2}\cdot 600^{2}}{32\cdot \sigma^{2}\cdot U^{2}}$$

where

*α* = Included angle (°);
*E* = Eccentricity;
*d* = Diameter of the cam plate;
*b* = Load ratio = γ1/γ2;
*σ* = Standard deviation of the Gaussion distribution;
*U* = Circumference of the cam plate.

**Figure 16:**
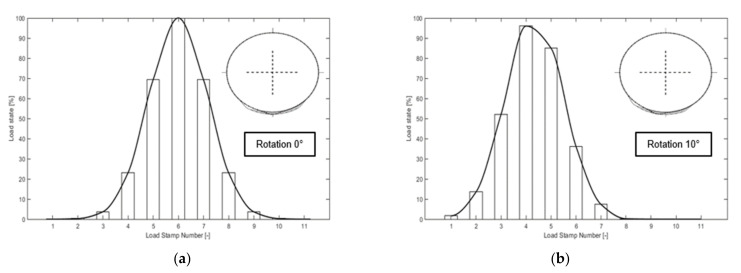
Load profile of load stamps at different instants. (**a**) α = 0°. (**b**) α = 10°.

The design of the cam geometry and arrangement allows the ARROWS to simulate the rolling motion of tire load on the pavement. Figure 15 presents the load applied to the asphalt slab at two instants, as an example. The location of all 11 cams is also shown in this figure. Figure 16a,b refer to the instant when the α of cam No. 6 rotates to 0° and 10°, respectively.

Benefiting from the driving mode of the ARROWS, the rotation speed of the shaft can be increased to a very high level (*v* = 80 km/h) without compromising the output performance. This means the ARROWS allows the researchers to evaluate the fatigue behavior of asphalt mixture under a realistic loading speed. Further advantages of the ARROWS testing device are the low effort of sample preparation, the automatic test execution, as well as comparably low costs for the acquisition and operation of the test device. The preparation of the specimens for testing with the ARROWS testing device is limited to the production of the asphalt specimens of the dimensions shown in 6.1. Further processing, such as cutting, drilling, or grinding of the specimens, which is required in other test methods, is not required. The device was designed in such a way that after manual specimen insertion, a fully automatic test is carried out, so that the correct execution of the load is ensured at all times. At the latest with the multiplication of the prototype of this concept, acquisition costs are expected to be in the range of comparable mechanically-based test systems, which are significantly lower than the costs for servo-hydraulic systems, as they are used for many fatigue tests.

## 7. Feasibility of ARROWS

**Figure 17:**
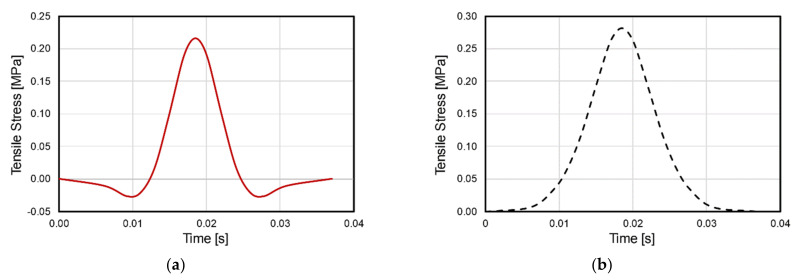
Horizontal stress at the bottom of asphalt slab. (**a**) Longitudinal stress. (**b**) Transverse stress.

The above-calculated load profiles are inputted into a FEM model to characterize the stress states of the asphalt mixture specimen loaded by ARROWS. In the FEM model, asphalt mixture slab and the rubber mat are surrounded by a steel container. The maximum vertical stress created by the stamp was set as 0.35 MPa, the stress of other stamps is then determined accordingly. The interface between the asphalt mixture and rubber layer was considered as frictionless. The modulus of the steel is set as 20 GPa. The material properties of the asphalt mixture are the same as that of the asphalt base course listed in Table 1. Because rubber only showed a tiny strain under the loading condition, the rubber was simplified as linear viscoelastic material which had a modulus of 10 MPa and a Poisson’s ratio of 0.49. Figure 17 shows the stress variation at the bottom center of the asphalt slab during a loading cycle, where a stress reversal is found in the longitudinal stress. In addition, the transverse stress is confirmed as a pure tensile stress during the loading cycle. Comparing Figure 17 with Figure 2 reveals that the ARROWS is capable of creating tensile stress, which is similar to that in the pavement structure, indicating the feasibility of using ARROWS to better simulate the realistic stress condition of asphalt mixture.

## 8. Findings and Conclusions

This paper starts by characterizing the tensile stress at bottom of a typical German asphalt pavement structure. After conducting a survey of more than 100 publications, the theoretical background, working mechanisms, test standards, and applications of the currently available fatigue test methods are discussed and summarized. As a solution to these limitations, an innovative fatigue test device, named ARROWS, is proposed. The following conclusions can be drawn from this work.

(1) The stress state in the lab specimens differs from that in the pavement. The tensile stress at the bottom of the asphalt layer is two-dimensional and contains a stress reversal at longitudinal direction. However, none of the available fatigue test methods are able to reproduce this 2D tensile stress.
(2) The experimental results and the theoretical analysis reveal that the load waveform and stress ratio strongly affect the fatigue performance of asphalt mixture. This highlights the importance of reproducing field stress conditions when evaluating the fatigue performance of asphalt mixture in the lab.
(3) The ARROWS is designed to better simulate the rolling motion of the tire load in the lab with a traffic speed using a special loading system. Specifically, vertical stress is applied to the test specimen via load stamps, which is connected to a shaft through a spring and a piston.
(4) The FEM model indicates the similarity between the stress at the bottom of the asphalt slab of the ARROWS specimen and that at the asphalt layer of pavement. This indicates the feasibility and the effectiveness of using the ARROWS as an alternative to the currently available fatigue test methods.

## Figures and Tables

**Table 1:** Information about pavement structure and materials.

Pavement Layer	Thickness [mm]	Elastic Modulus [MPa]	Poisson’s Ratio
Surface course	40	5581	0.27
Asphalt binder course	80	9686	0.27
Asphalt base course	220	6481	0.27
Frost protection layer	510	325	0.35
Subgrade	2000	50	0.40

**Table 2:** Summary of fatigue test methods.

Test	Test Specification	Loading Type	Measured Material Properties	Failure Occurs in a Uniform Stress/Strain Zone?	Stress Characteristics	Stress Reversal?
4PB	AASHTO T321-14 ASTM D8237-18 EN 12697-24:2018	Strain-controlled sinusoidal loading wave	Stress, phase angle	Yes	1D *	Yes
3PB	-	Strain-/stress controlled haversine loading wave	Stress/strain, phase angle	No	1D	No
2PB	EN 12697-24:2018	Force-/displacement-controlled sinusoidal load	Displacement	No	1D	Yes
Axial fatigue test	AASHTO TP107	Force-/displacement-controlled	Stress/strain, phase angle	Yes	1D	Yes (TCFT)
Loaded wheel fatigue test	-	Moving wheel load	Strain at sample bottom, sample deformation	No	1D	Yes
ITFT	EN 12697-24:2018	Sinusoidal/haversine	Strain	No	2D	No
Shear fatigue test	-	Sinusoidal load	Stress/strain, phase angle	Yes	1D	Yes

- test specification is not available because the test method is not a standard fatigue test method. * D refers to dimension(s).

**Table 3:** Relationship between vehicle speed and loading time (*t*) or loading frequency (*f*).

Authors	Loading Time (t) or Loading Frequenzy (f)	Remark
NCHRP [101]	t = L_eff_/17.6v_s_	L_eff_ = effective length
Barksdale [102]	22.7 Hz	v = 72 km/h; depth: 30.5 cm
Mollenhauer et al. [103]	f = 0.277 v^0.944^	*-*

## Data Availability

Not applicable.
